# Characterizing Musculoskeletal and Neurological Toxicities Associated With the BPaLM Regimen: A Clinical Evaluation of Arthralgia and Peripheral Neuropathy in Patients With Multidrug-Resistant Tuberculosis (MDR-TB)

**DOI:** 10.7759/cureus.86248

**Published:** 2025-06-17

**Authors:** Zahir Khan, Gohar Ali, Rumman, Ashraf, Akmal Naveed, Shahid Salam, Ubaid Ullah, Ahmad Ismail, Afrasyab Altaf

**Affiliations:** 1 Orthopedic Surgery, Medical Teaching Institution (MTI) Mardan Medical Complex, Bacha Khan Medical College, Mardan, PAK; 2 Neurosurgery, Medical Teaching Institution (MTI) Mardan Medical Complex, Bacha Khan Medical College, Mardan, PAK; 3 Pharmacovigilance/Active Drug Safety Monitoring and Management, Medical Teaching Institution (MTI) Mardan Medical Complex, Mardan, PAK; 4 Pharmacovigilance/Active Drug Safety Monitoring and Management, Association for Community Development, Peshawar, PAK; 5 Research and Development, Pro-Gene Diagnostics and Research Laboratory, Mardan, PAK; 6 Epidemiology and Public Health, Association for Community Development, Peshawar, PAK; 7 Pulmonology, Provincial Tuberculosis Control Program, Directorate of Health, Peshawar, PAK; 8 Tuberculosis, Association For Community Development, Peshawar, PAK; 9 Pulmonology, Medical Teaching Institution (MTI) Mardan Medical Complex, Mardan, PAK; 10 Epidemiology and Public Health, National Tuberculosis Control Program, Ministry of National Health Services, Regulations, and Coordination, Islamabad, PAK; 11 Cardiology, Rehman Medical Institute, Peshawar, PAK

**Keywords:** arthralgia, bpalm regimen, mdr-tb, musculoskeletal toxicities, neurological toxicities, patient management, peripheral neuropathy

## Abstract

Background: Musculoskeletal and neurological toxicities are common side effects of the BPaLM (bedaquiline, pretomanid, linezolid, and moxifloxacin) regimen, an emerging treatment for multidrug-resistant tuberculosis (MDR-TB). These toxicities, particularly arthralgia and peripheral neuropathy, can significantly impair the quality of life of patients undergoing treatment. Despite the promising therapeutic benefits of the BPaLM regimen, the prevalence and severity of these side effects remain underexplored. Understanding these toxicities is crucial to improving patient management strategies and ensuring better treatment adherence.

Objective: This study aims to determine how common and severe musculoskeletal and neurological toxicities, particularly arthralgia and peripheral neuropathy, are among MDR-TB patients treated with the BPaLM regimen.

Materials and methods: This prospective observational study was conducted at the Programmatic Management of Drug-Resistant Tuberculosis in Mardan Medical Complex between January 2024 and April 2025. Patients with MDR-TB undergoing treatment with the BPaLM regimen were monitored for musculoskeletal and neurological toxicities, specifically arthralgia and peripheral neuropathy. Clinical evaluations included assessing the onset, severity, and impact of joint pain and nerve damage, as well as evaluating the effectiveness of pain management and physical therapy interventions. Data collection included demographic information, comorbidities, and baseline physical activity levels. Statistical analysis was performed using SPSS Statistics version 26 (IBM Corp. Released 2019. IBM SPSS Statistics for Windows, Version 26.0. Armonk, NY: IBM Corp.), Python (Python Software Foundation, Beaverton, OR, USA), and R 4.4.5 (R Foundation for Statistical Computing, Vienna, Austria) to identify significant predictors of toxicity severity through descriptive statistics, chi-square tests, and decision tree modeling. Kaplan-Meier survival analysis was also conducted to assess the relationship between toxicity severity and treatment outcomes.

Results: Among the 44 MDR-TB patients, 35 (79.54%) experienced mild to moderate arthralgia, with knee pain being most common (34, 77.27%). Peripheral neuropathy was reported in 26 (59.09%) patients, with the lower limbs (20, 45.45%) being most affected. Kaplan-Meier survival analysis revealed a significant difference in survival times based on the severity of arthralgia and peripheral neuropathy, with more severe symptoms correlating with reduced survival duration.

Conclusions: The findings underscore the importance of early identification, regular monitoring, and personalized management strategies to mitigate the burden of these toxicities and enhance patient outcomes.

## Introduction

Multidrug-resistant tuberculosis (MDR-TB) remains a critical global health issue, particularly in regions with high TB prevalence and limited access to effective treatments. MDR-TB refers to *Mycobacterium tuberculosis* strains resistant to at least two of the most potent first-line anti-TB drugs: isoniazid and rifampicin. The development of MDR-TB is primarily associated with improper use of antimycobacterial medications, inadequate treatment protocols, and poor patient adherence [1,2]. This not only exacerbates individual health outcomes but also presents significant public health risks, especially in developing countries with limited healthcare resources [3,4].

Advances in treatment regimens, such as the BPaLM (bedaquiline, pretomanid, linezolid, and moxifloxacin) regimen, offer hope for combating MDR-TB. Each drug in the combination targets resistant strains of TB through distinct mechanisms. Bedaquiline inhibits ATP synthase in *Mycobacterium tuberculosis*, preventing bacterial growth [5,6]. Pretomanid, a nitroimidazole, is effective against various resistant strains, while linezolid (an oxazolidinone) and moxifloxacin (a fluoroquinolone) provide coverage by penetrating bacterial cell membranes [7,8]. These therapies have significantly improved treatment success rates for MDR-TB patients.

However, the BPaLM regimen is not without side effects. Common toxicities include musculoskeletal and neurological issues such as arthralgia and peripheral neuropathy. Arthralgia, which causes joint pain, can hinder mobility, while peripheral neuropathy, characterized by tingling and numbness, significantly affects patients' quality of life [9,10]. These adverse effects may lead to treatment non-adherence or premature discontinuation, emphasizing the need for further research into their management.

Studies have documented peripheral neuropathy associated with linezolid, potentially resulting from drug-induced mitochondrial dysfunction, which is vital for neuronal function [11,12]. However, there is limited research on the full scope of side effects caused by the BPaLM regimen. Therefore, further investigation is essential to assess the prevalence and severity of these toxicities, ensuring a balanced approach between treatment efficacy and patient welfare.

The global burden of MDR-TB continues to rise, underscoring the need for detailed safety assessments of regimens like BPaLM. Many patients experience side effects that lead to deviations from prescribed therapies, affecting treatment outcomes [4,13]. Factors such as age, co-infections, and clinical history must be considered when managing MDR-TB treatment [14,15]. Success rates for MDR-TB patients are generally lower than for drug-sensitive TB due to the complexity of individual responses to therapy [16,17].

This study aims to systematically assess the prevalence and severity of musculoskeletal and neurological toxicities in patients receiving the BPaLM regimen. Through both qualitative and quantitative methods, we aim to gain a deeper understanding of these side effects and inform more effective management practices for MDR-TB patients [18,19]. By addressing the safety concerns associated with potent treatments like BPaLM, we aim to enhance patient support systems and treatment adherence.

Given the rising incidence of MDR-TB, particularly in underserved populations, balancing therapeutic efficacy with side-effect management is critical. Establishing guidelines for monitoring adverse events associated with the BPaLM regimen is crucial for enhancing patient outcomes and protecting their welfare [20,21].

Through collaboration, clinicians can develop personalized treatment strategies to mitigate adverse effects like arthralgia and peripheral neuropathy, ultimately enhancing health outcomes for MDR-TB patients [22,23]. This study aims to contribute to the evolving landscape of MDR-TB treatment, highlighting the need for ongoing improvement in clinical approaches.

## Materials and methods

Study design

This quantitative, observational study was conducted at the Department of Programmatic Management of Drug-Resistant Tuberculosis, Mardan Medical Complex, from January 2024 to April 2025. The study aimed to evaluate the musculoskeletal and neurological toxicities associated with the BPaLM regimen in patients with MDR-TB, specifically focusing on arthralgia (joint pain) and peripheral neuropathy (nerve damage). A prospective data collection approach was employed, with regular assessments conducted throughout the treatment regimen. Table 1 shows the inclusion and exclusion criteria.

**Table 1:** Inclusion and exclusion criteria MDR-TB: multidrug-resistant tuberculosis, BPaLM: bedaquiline, pretomanid, linezolid, and moxifloxacin

Criteria type	Criterion
Inclusion	Patients of any age diagnosed with MDR-TB
Inclusion	Patients undergoing treatment with the BPaLM regimen
Inclusion	Patients who provided informed consent to participate in the study
Exclusion	Patients with pre-existing musculoskeletal or neurological disorders unrelated to MDR-TB treatment
Exclusion	Pregnant or breastfeeding women
Exclusion	Patients with severe co-morbidities (e.g., chronic renal failure, liver disease, cardiovascular disease) that could interfere with the study's outcomes
Exclusion	Patients receiving treatment for active infections other than MDR-TB

Sampling method

A convenience sampling approach was employed to select eligible patients from the Orthopedics Department at Mardan Medical Complex. Participants were stratified by age, gender, and baseline disease severity.

Sample Size Calculation

The sample size for this study was calculated based on a population of 44 patients, with a 95% confidence level and a 5% margin of error [24]. The required sample size for detecting statistically significant differences in toxicity outcomes was approximately 40 patients, ensuring the study had an adequate sample size for reliable results. Therefore, the inclusion of 44 patients in the study was sufficient for statistical analysis.

Data Collection

Data were gathered using a combination of patient questionnaires and clinical evaluations. At baseline, demographic details, including age, gender, BMI, smoking history, and physical activity level, were collected, along with information on comorbidities. Arthralgia (joint pain) was assessed by recording the onset, severity, location, functional disability, pain response to analgesics, impact on quality of life, and severity of tendonitis. Neuropathy was evaluated by noting the onset, severity, distribution of symptoms, muscle weakness, balance and coordination, and sensory nerve function. Patients were followed throughout their treatment with the BPaLM regimen, with particular attention to their response to pain treatment and any physical therapy interventions used to alleviate symptoms.

Data analysis

All statistical analyses were carried out using SPSS Statistics version 26 (IBM Corp. Released 2019. IBM SPSS Statistics for Windows, Version 26.0. Armonk, NY: IBM Corp.) for data analysis, while Python (Python Software Foundation, Beaverton, OR, USA) and R 4.4.5 (R Foundation for Statistical Computing, Vienna, Austria) libraries were utilized for supplementary graphical visualizations. Any missing data were addressed using imputation techniques to minimize bias. Missing values were imputed based on the observed patterns in the dataset, ensuring minimal disruption to the statistical analysis. Descriptive statistics were applied to summarize the data, with continuous variables presented as mean ± standard deviation (SD) and categorical variables summarized using frequencies and percentages. A bar plot and chi-square tests were employed to examine the relationships between various factors, including gender, smoking history, baseline physical activity, comorbidities, frequency of musculoskeletal pain, functional disability, response to pain medications, physical therapy usage, tendon involvement, impact on balance and coordination, response to neuropathic pain treatment, muscle weakness, sensory nerve function, and neuropathy distribution, in relation to the presence and severity of neuropathy and arthralgia. The Kruskal-Wallis test was applied to compare age distribution and BMI across different levels of neuropathy and arthralgia severity. Kaplan-Meier survival analysis was utilized to estimate survival curves and evaluate the influence of arthralgia and neuropathy severity on treatment outcomes. Decision tree analysis using the Classification and Regression Trees algorithm was performed to model the predictors of arthralgia and neuropathy severity, with the Gini index used to determine the significance of each predictor, such as age and BMI. Raincloud plots were used to investigate the distribution of baseline physical activity levels, as well as the presence and severity of neuropathy and arthralgia, in relation to age and BMI. Feature importance analysis was conducted using machine learning algorithms, specifically the random forest model, to assess the impact of all studied variables on the severity of arthralgia and neuropathy. A p-value of <0.05 was considered statistically significant.

Ethical considerations

The study was conducted in full compliance with the ethical guidelines approved by the Institutional Review Board of the Association for Community Development, Khyber Pakhtunkhwa, with approval reference number 101/ERC/ACD. All participants provided written informed consent before participation, confirming their understanding of the study's purpose, procedures, and potential risks. Confidentiality was maintained throughout the study, with all data anonymized and securely stored. No additional financial or medical burdens were imposed on participants.

## Results

A total of 44 MDR-TB patients were included in the study, consisting of 20 males (45.45%) and 24 females (54.55%). The mean age of the participants was 32.49 ± 17.75 years, with an average BMI of 17.9 ± 3.34. A smoking history was reported by six patients (13.64%). Regarding baseline physical activity, six patients (13.64%) were categorized as highly active, 26 patients (59.09%) as moderately active, and 12 patients (27.27%) as sedentary. Additionally, six patients (13.64%) had comorbidities, with diabetes being the most prevalent (Table 2).

**Table 2:** Demographic and baseline characteristics of MDR-TB patients Data is presented as frequency and percentage or mean and standard deviation. MDR-TB: multidrug-resistant tuberculosis, BMI: body mass index

Characteristics	Values
Total patients	44 (100%)
Gender	
Male	20 (45.45%)
Female	24 (54.55%)
Age	32.49 ± 17.75
BMI	17.9 ± 3.34
Smoking history	06 (13.64%)
Baseline physical activity level	
Highly active	06 (13.64%)
Moderately active	26 (59.09%)
Sedentary	12 (27.27%)
Comorbidities	
Diabetes	06 (13.64%)

Table 3 presents the arthralgia-related characteristics of the 44 MDR-TB patients treated with the BPaLM regimen. No patients reported a family history of musculoskeletal disorders or previous musculoskeletal issues. The average onset of joint pain occurred 90.27 ± 48.97 days after the start of treatment. Regarding the presence and severity of arthralgia, seven patients (15.91%) experienced no pain, while 26 patients (59.09%) reported mild pain, nine patients (20.45%) moderate pain, and two patients (4.55%) severe pain. Joint pain was most commonly located in the knees (77.27%), followed by ankles (18.18%), arms (4.55%), shoulders (6.82%), and wrists (6.82%). Functional disability was absent in 32 patients (72.73%), mild in 10 patients (22.73%), and moderate in two patients (4.55%). Regarding pain response to analgesics, seven patients (15.91%) required no medication, 33 patients (75.00%) experienced complete pain relief, and four patients (9.09%) had partial relief. In terms of the duration and frequency of musculoskeletal pain, seven patients (15.91%) reported no pain, 28 patients (63.24%) had occasional pain, and nine patients (20.45%) experienced frequent pain. The impact of musculoskeletal symptoms on quality of life was reported as having no impact in seven patients (15.91%), mild impact in 26 patients (59.09%), moderate impact in 10 patients (22.73%), and severe impact in one patient (2.27%). Physical therapy intervention was used by 13 patients (29.55%), with 31 patients (70.45%) requiring no therapy. Tendon involvement was noted in 15 patients (34.09%), with the Achilles tendon being most commonly affected. Tendonitis severity was mild in 10 patients (22.72%), moderate in four patients (9.09%), and severe in one patient (2.27%).

**Table 3:** Arthralgia-related characteristics in MDR-TB patients treated with the BPaLM regimen Data is presented as frequency and percentage or mean and standard deviation. MDR-TB: multidrug-resistant tuberculosis, BPaLM: bedaquiline, pretomanid, linezolid, and moxifloxacin

Characteristics	Values
Total patients	44 (100%)
Family history of musculoskeletal disorders	00 (00.00%)
Previous musculoskeletal issues	00 (00.00%)
Onset of joint pain (days after tx start)	90.27 ± 48.97
Presence and severity of arthralgia	
No	07 (15.91%)
Mild	26 (59.09%)
Moderate	09 (20.45%)
Severe	02 (4.55%)
Location of joint pain	
Nil	07 (15.91%)
Knees	34 (77.27%)
Ankles	08 (18.18%)
Arm	02 (4.55%)
Shoulder	03 (6.82%)
Wrist	03 (6.82%)
Functional disability	
None	32 (72.73%)
Mild	10 (22.73%)
Moderate	02 (4.55%)
Pain response to analgesics	
No medication needed	07 (15.91%)
Complete pain relief	33 (75.00%)
Partial relief	04 (9.09%)
Duration and frequency of musculoskeletal pain	
No pain	07 (15.91%)
Occasional	28 (63.24%)
Frequent	09 (20.45%)
Impact of musculoskeletal symptoms on quality of life	
No impact	07 (15.91%)
Mild	26 (59.09%)
Moderate	10 (22.73%)
Severe	01 (2.27%)
Physical therapy use or intervention	
None	31 (70.45%)
Mild	03 (6.81%)
Moderate	10 (22.72%)
Tendon Involved	
None	29 (65.91%)
Achilles tendon	15 (34.09%)
Severity of Tendonitis	
None	29 (65.91%)
Mild	10 (22.72%)
Moderate	04 (9.09%)
Severe	01 (2.27%)

Table 4 outlines the neuropathy-related characteristics of the 44 MDR-TB patients treated with the BPaLM regimen. None of the patients reported a family history of neurological disorders or previous neurological issues. All patients (100%) demonstrated normal cognitive function. The average onset of neuropathy occurred 110.76 ± 42.88 days after the start of treatment. Regarding the presence and severity of peripheral neuropathy, 18 patients (40.91%) reported no neuropathy, while 18 patients (40.91%) had mild neuropathy, six patients (13.64%) had moderate neuropathy, and two patients (4.55%) had severe neuropathy. Neuropathy was most commonly distributed in the lower limbs (45.45%), followed by the feet (34.09%), upper limbs (11.36%), and hands (15.91%). The severity of paresthesia was nil in 18 patients (40.91%), mild in 18 patients (40.91%), moderate in six patients (13.64%), and severe in two patients (4.55%). Muscle weakness was observed with varying resistance, with four patients (9.09%) experiencing weakness throughout the full range against gravity, 28 patients (63.64%) experiencing weakness against some resistance, and 12 patients (27.27%) experiencing weakness against strong resistance. The impact of neuropathy on balance and coordination was minimal, with 36 patients (81.82%) reporting no impact, six patients (13.64%) reporting mild impact, and two patients (4.55%) reporting moderate impact. Sensory nerve function was normal in 18 patients (40.91%), mildly impaired in 21 patients (47.73%), and moderately impaired in five patients (11.36%). In terms of response to neuropathic pain treatment, 18 patients (40.91%) required no treatment, three patients (6.82%) had a partial response, and 23 patients (52.27%) experienced a full response. Physical therapy use was reported by 13 patients (29.55%), with 31 patients (70.45%) requiring no therapy.

**Table 4:** Neuropathy-related characteristics in MDR-TB patients treated with the BPaLM regimen Data is presented as frequency and percentage or mean and standard deviation. MDR-TB: multidrug-resistant tuberculosis, BPaLM: bedaquiline, pretomanid, linezolid, and moxifloxacin

Characteristics	Values
Total patients	44 (100%)
Family history of neurological disorders	00 (00.00%)
Previous neurological issues	00 (00.00%)
Normal cognitive function	44 (100%)
Onset of neuropathy (from start of tx in days)	110.76 ± 42.88
Presence and severity of peripheral neuropathy	
No neuropathy	18 (40.91%)
Mild	18 (40.91%)
Moderate	06 (13.64%)
Severe	02 (4.55%)
Neuropathy distribution	
None	18 (40.91%)
Feet	22 (50%)
Lower limbs	20 (45.45%)
Upper limb	05 (11.36%)
Severity of paresthesia	
Nil	18 (40.91%)
Mild	18 (40.91%)
Moderate	06 (13.64%)
Severe	02 (4.55%)
Muscle weakness	
Through full range actively against gravity	04 (9.09%)
Through full range actively against some resistance	28 (63.64%)
Through full range actively against strong resistance	12 (27.27%)
Impact on balance and coordination	
No impact	36 (81.82%)
Mild	06 (13.64%)
Moderate	02 (4.55%)
Sensory nerve function	
Normal	18 (40.91%)
Mild impairment	21 (47.73%)
Moderate impairment	05 (11.36%)
Response to neuropathic pain treatment	
No need for treatment	18 (40.91%)
Partial response	03 (6.82%)
Full response	23 (52.27%)
Physical therapy use	
None	31 (70.45%)
Mild	03 (6.82%)
Moderate	10 (22.73%)

**Figure 1:**
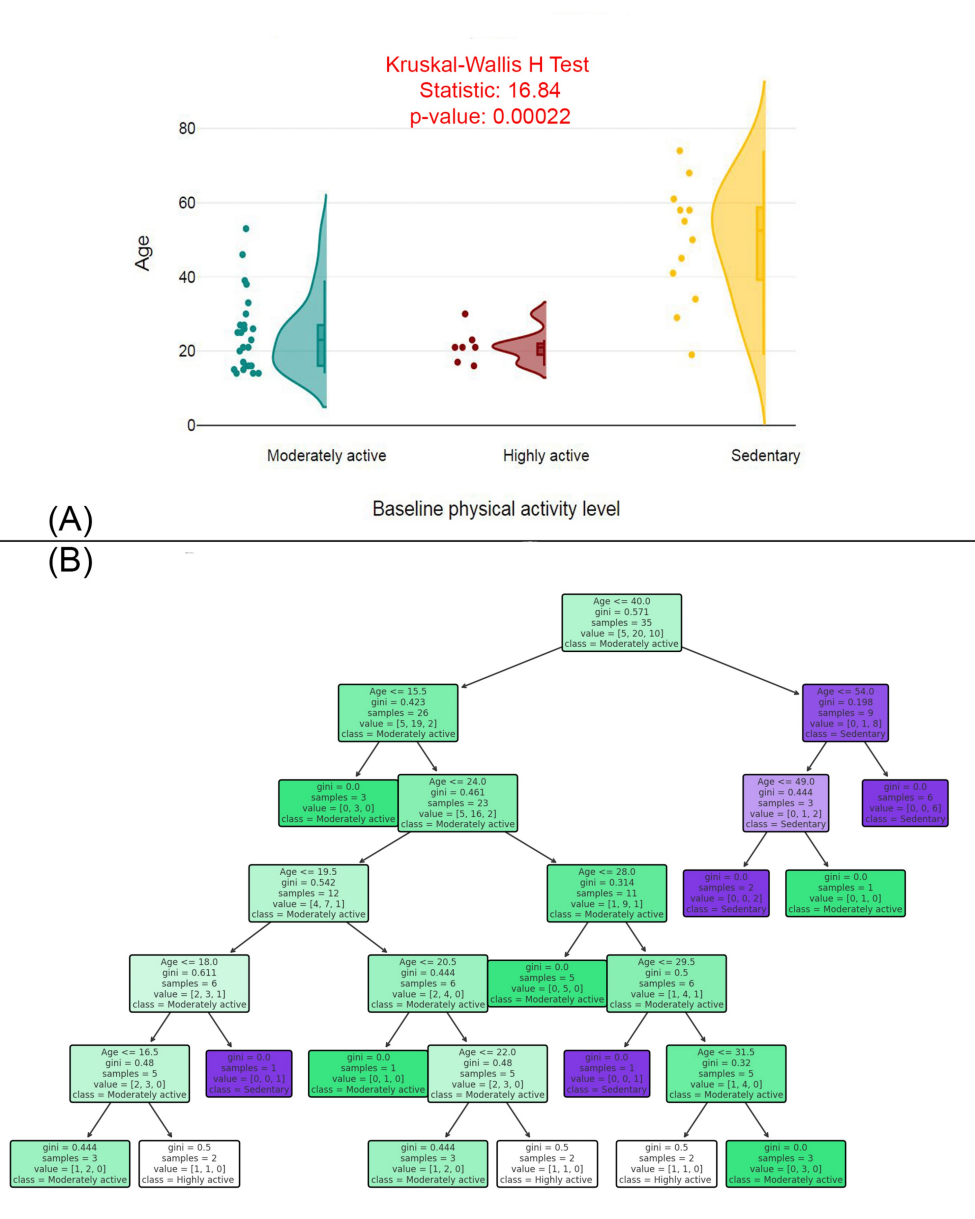
Age-related differences in physical activity levels: statistical and predictive analysis (A) Age distribution by baseline physical activity level. (B) Decision tree model predicting physical activity level based on age.

Figure 1A features a raincloud plot comparing age distributions across different baseline physical activity levels (moderately active, highly active, and sedentary), with a Kruskal-Wallis H Test statistic of 16.84 and a p-value of 0.00022, indicating a significant difference in age across the groups. The plot shows that the "moderately active" group is younger and has a wider age range compared to the "highly active" and "sedentary" groups, which have older and more limited age distributions. Figure 1B displays a decision tree model that predicts physical activity levels based on age, with Gini impurity values indicating the level of homogeneity within each group. The decision tree reveals key age thresholds, such as ≤40.0 and ≤15.5, that are used to classify individuals into "moderately active," "highly active," or "sedentary" categories. The age distribution significantly differs among activity levels, and the decision tree offers a predictive framework for classifying physical activity based on age.

**Figure 2:**
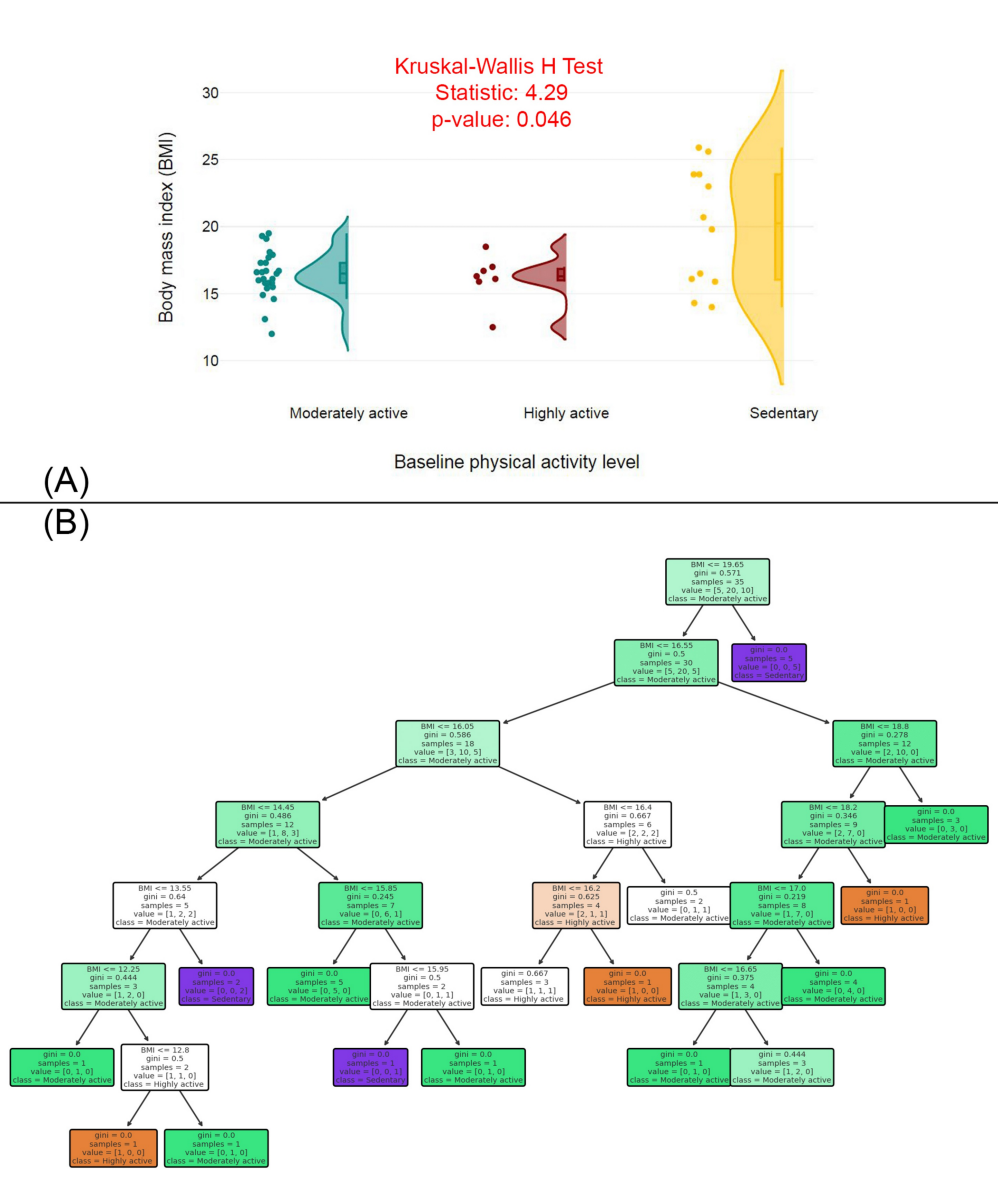
BMI and predictive modeling of physical activity levels (A) BMI distribution by baseline physical activity level. (B) Decision tree model predicting physical activity level based on BMI. BMI: body mass index

Figure 2A features a raincloud plot comparing the BMI distributions across three baseline physical activity levels (moderately active, highly active, and sedentary). The Kruskal-Wallis H Test statistic is 4.29, and the p-value is 0.046, suggesting a statistically significant difference in BMI between the three groups. The plot shows that individuals in the "moderately active" group have a wider range of BMI values compared to the "highly active" and "sedentary" groups. The "sedentary" group has a more concentrated, lower BMI distribution, while the "highly active" group has a more varied range of BMIs. Figure 2B illustrates a decision tree model for predicting physical activity levels based on BMI. The decision tree splits the data based on BMI thresholds, with Gini impurity values shown at each decision node, indicating the homogeneity of the resulting groups. The decision tree suggests key BMI thresholds, such as a BMI of ≤16.05 and ≤14.85, to classify individuals as "moderately active" or "highly active."

**Figure 3:**
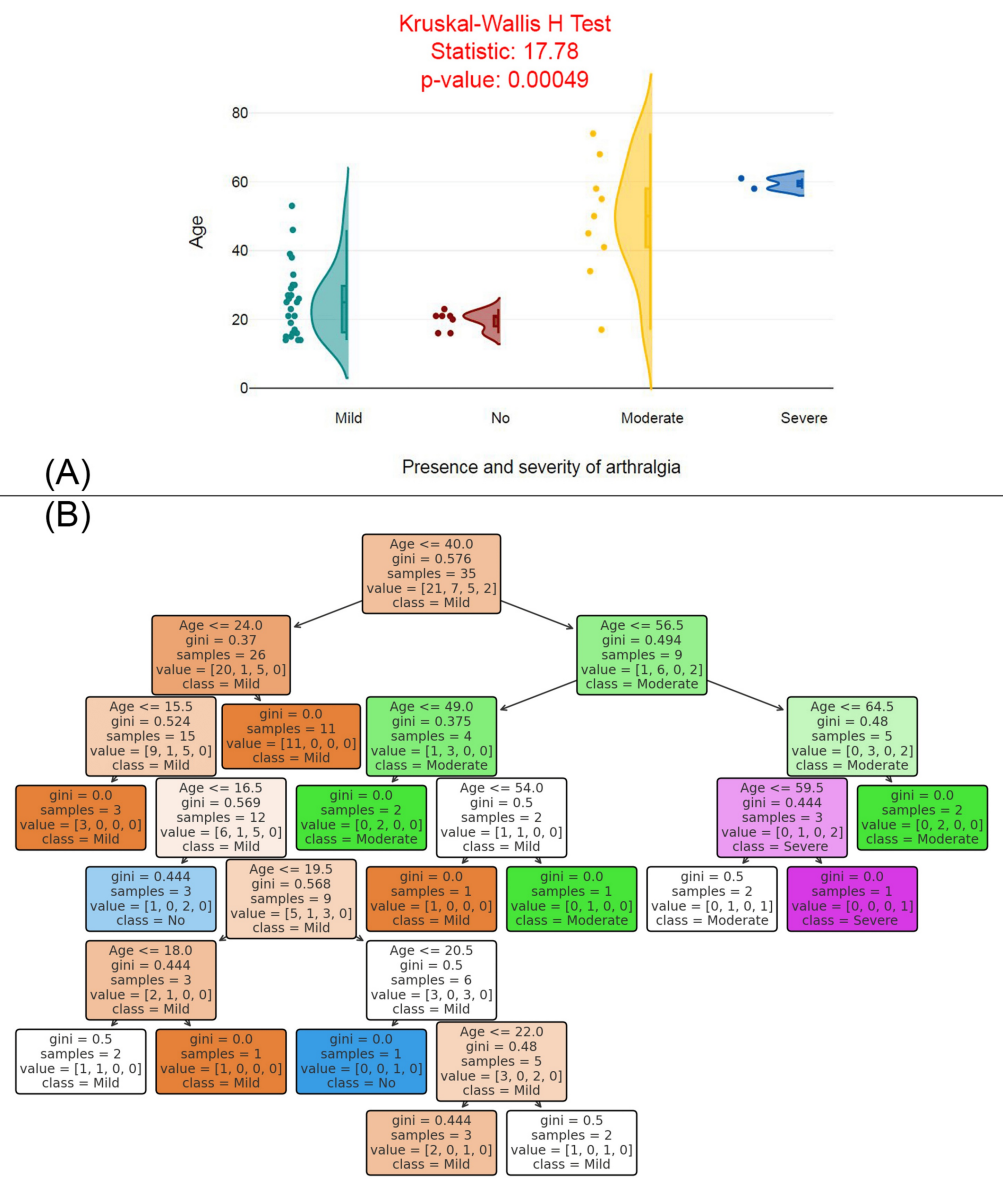
Age distribution and predictive modeling of arthralgia severity (A) Age distribution by presence and severity of arthralgia. (B) Decision tree model predicting arthralgia severity based on age.

Figure 3A shows a raincloud plot comparing the age distributions among different severity levels of arthralgia (no, mild, moderate, severe). The Kruskal-Wallis H Test statistic is 17.78, with a p-value of 0.00049, indicating a statistically significant difference in age across the severity levels of arthralgia. The plot suggests that individuals with more severe arthralgia (moderate and severe groups) tend to be older, with a more concentrated age range. In contrast, individuals with mild arthralgia and no arthralgia are generally younger, with a wider distribution of ages. Figure 3B displays a decision tree model predicting the severity of arthralgia based on age. The decision tree splits individuals based on key age thresholds, with Gini impurity values at each node indicating the level of homogeneity within the resulting groups. For example, individuals under age 40 are more likely to have mild arthralgia, while those over age 56.5 are more likely to experience moderate or severe arthralgia. The decision tree uses these age thresholds to classify individuals into different severity levels of arthralgia.

**Figure 4:**
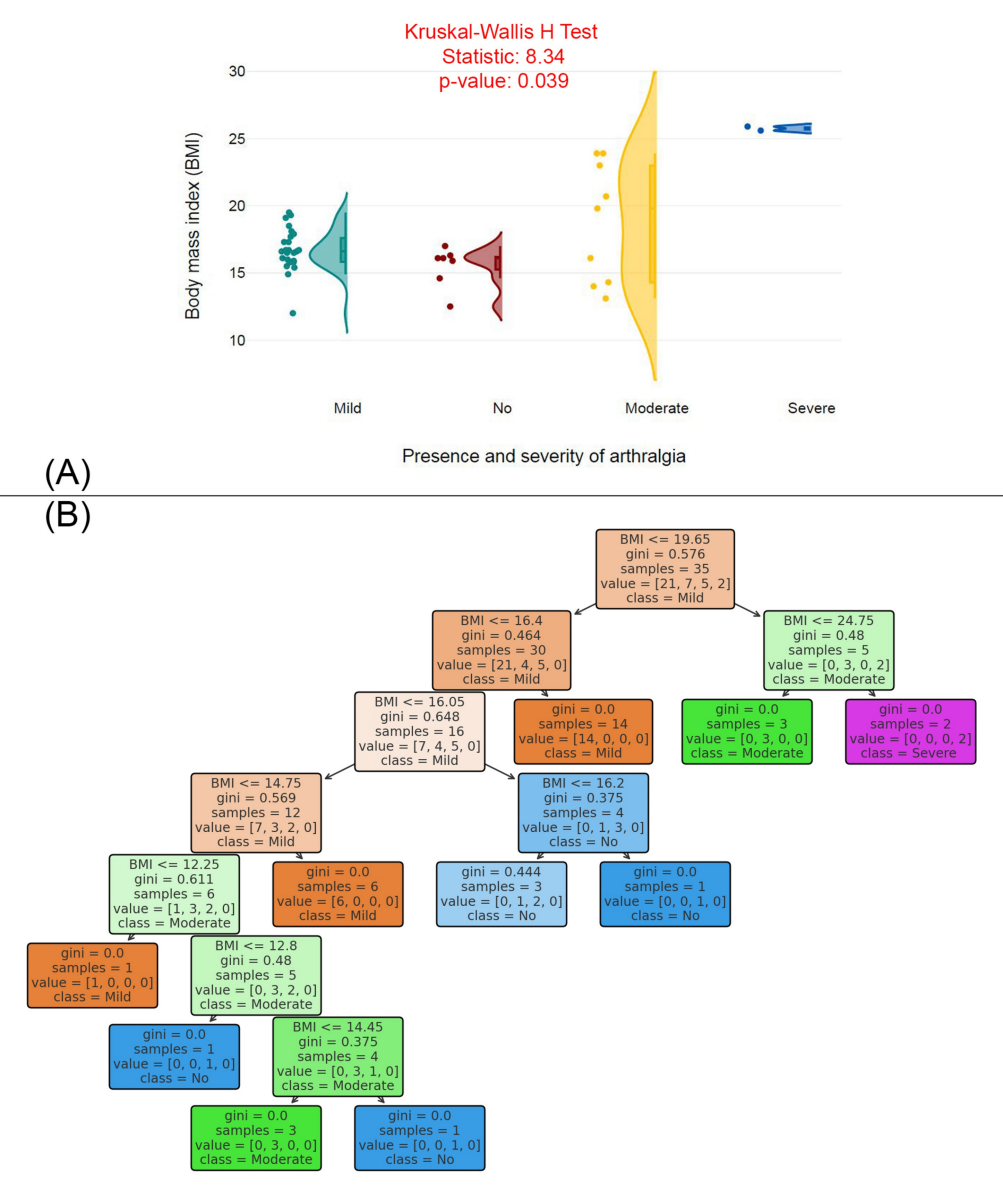
BMI and predictive modeling of arthralgia severity (A) BMI distribution by presence and severity of arthralgia. (B) Decision tree model predicting arthralgia severity based on BMI. BMI: body mass index

Figure 4A features a violin plot comparing BMI distributions across different severity levels of arthralgia (mild, no, moderate, severe). The Kruskal-Wallis H Test statistic is 8.34, and the p-value is 0.039, indicating a statistically significant difference in BMI between the groups. The plot shows that individuals with more severe arthralgia (moderate and severe) tend to have a higher BMI compared to those with mild or no arthralgia, with a more concentrated BMI range in the moderate and severe groups. Figure 4B displays a decision tree model that predicts the severity of arthralgia based on BMI. The decision tree splits individuals based on BMI thresholds, with Gini impurity values at each node indicating the level of homogeneity in the resulting groups. Key BMI thresholds, such as ≤16.05, ≤19.65, and ≤24.75, are used to classify individuals into different levels of arthralgia severity (mild, moderate, no, and severe).

**Figure 5:**
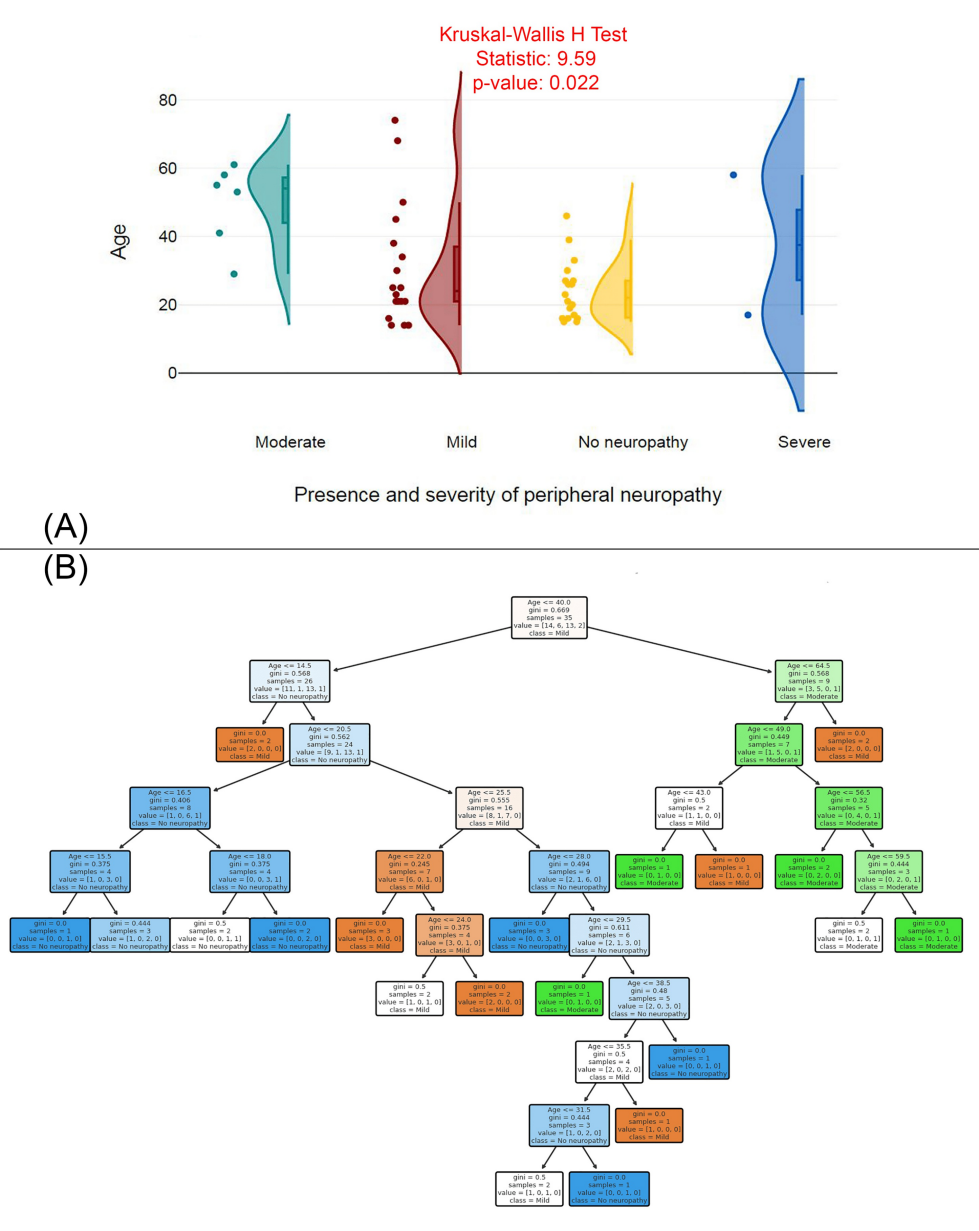
Age distribution and predictive modeling of peripheral neuropathy severity (A) Age distribution by presence and severity of peripheral neuropathy. (B) Decision tree model predicting peripheral neuropathy severity based on age.

Figure 5A displays a raincloud plot comparing the age distributions across different levels of peripheral neuropathy severity (moderate, mild, no neuropathy, severe). The Kruskal-Wallis H Test statistic is 9.59, with a p-value of 0.022, indicating a statistically significant difference in age between the groups. The plot suggests that individuals with more severe neuropathy (moderate and severe) tend to be older, with a more concentrated age range. In contrast, the "no neuropathy" group comprises younger individuals with a wider age range. Figure 5B shows a decision tree model that predicts the presence and severity of peripheral neuropathy based on age. The decision tree splits the data based on specific age thresholds, with Gini impurity values indicating the level of homogeneity within the resulting groups. Key age thresholds, such as ≤16.5, ≤25.5, and ≤35.5, are used to classify individuals into the different severity levels of peripheral neuropathy (mild, no neuropathy, moderate, severe).

**Figure 6:**
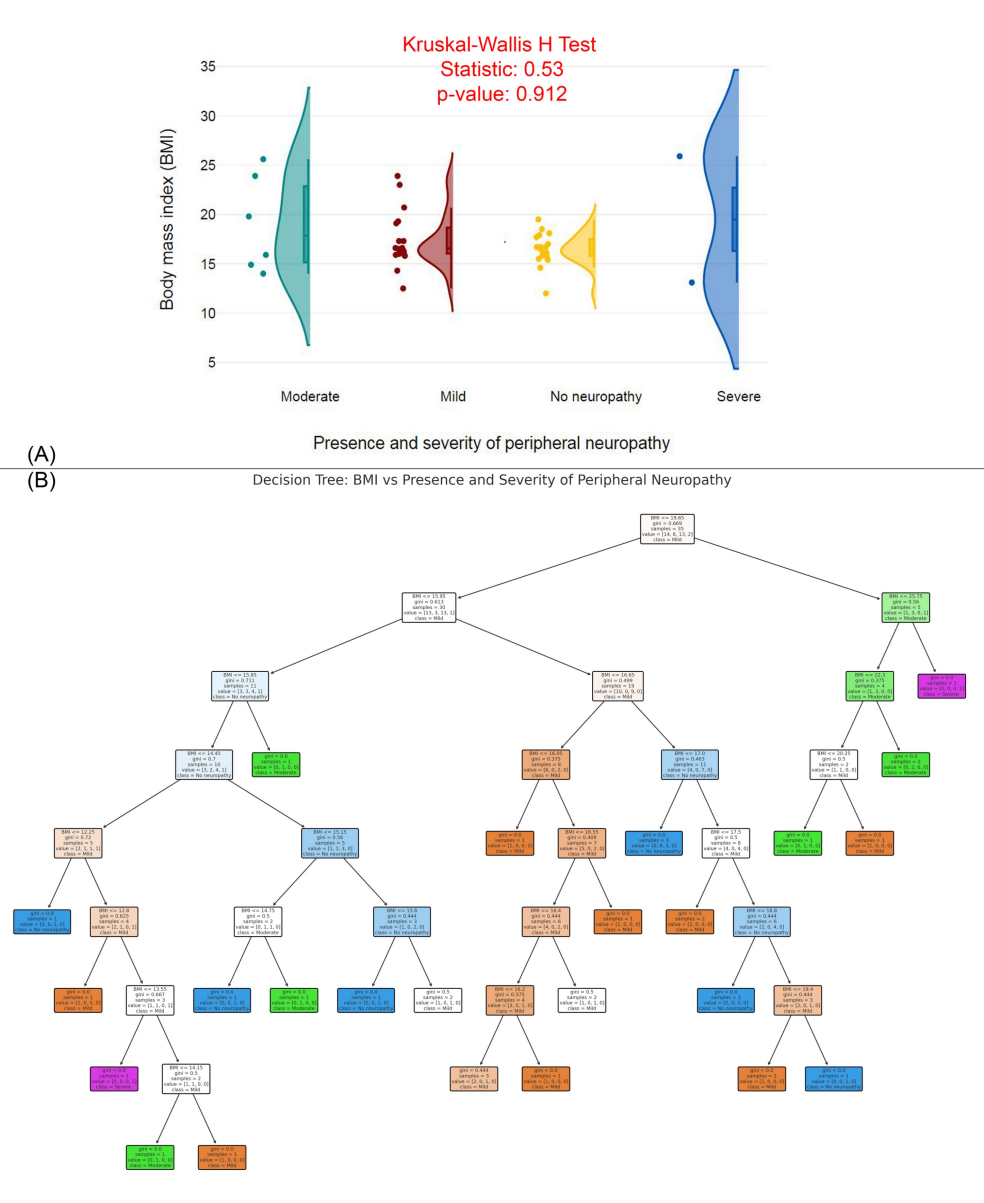
BMI and predictive modeling of peripheral neuropathy severity (A) BMI distribution by presence and severity of peripheral neuropathy. (B) Decision tree model predicting peripheral neuropathy severity based on BMI. BMI: body mass index

Figure 6A features a raincloud plot comparing the BMI distributions across different levels of peripheral neuropathy severity (moderate, mild, no neuropathy, severe). The Kruskal-Wallis H Test statistic is 0.53, with a p-value of 0.912, indicating that there is no significant difference in BMI across the groups. The plot shows relatively similar BMI distributions across the four groups, with some overlap in the ranges. Figure 6B presents a decision tree model predicting the presence and severity of peripheral neuropathy based on BMI. The decision tree splits individuals based on specific BMI thresholds, with Gini impurity values showing the homogeneity of the resulting groups. This model predicts neuropathy severity using BMI, but given the lack of significant findings from the Kruskal-Wallis test in Figure 6A, it suggests that BMI may not be a strong predictor of neuropathy severity in this case.

**Figure 7:**
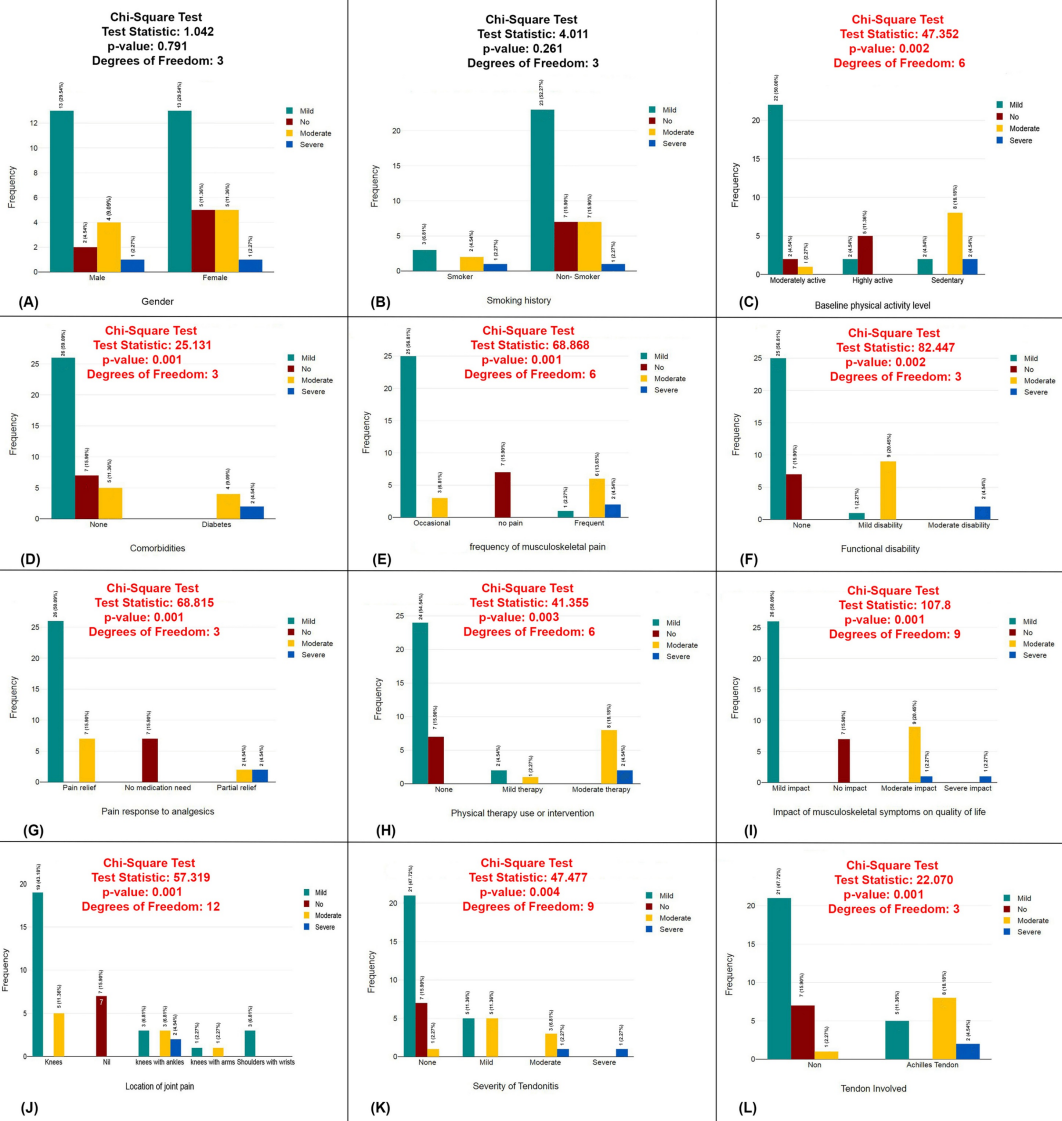
Chi-square analysis of factors associated with the severity of arthralgia (A) Gender distribution by severity. (B) Smoking history by severity. (C) Baseline physical activity level by severity. (D) Comorbidities by severity. (E) Frequency of musculoskeletal pain by severity. (F) Functional disability by severity. (G) Pain response to analgesics by severity. (H) Physical therapy intervention by severity. (I) Impact of musculoskeletal pain on quality of life by severity. (J) Location of joint pain by severity. (K) Severity of tendinitis by severity. (L) Tendon involvement by severity.

The plots present chi-square tests analyzing various factors associated with the severity of arthralgia (mild, moderate, severe). Significant relationships (p<0.05) were found between the severity of the condition and several variables, including baseline physical activity level, comorbidities, frequency of musculoskeletal pain, functional disability, pain response to analgesics, physical therapy interventions, the impact of pain on quality of life, joint pain location, severity of tendinitis, and tendon involvement, all with p-values of 0.001-0.004. However, no significant associations were observed for gender or smoking history (Figure 7).

**Figure 8:**
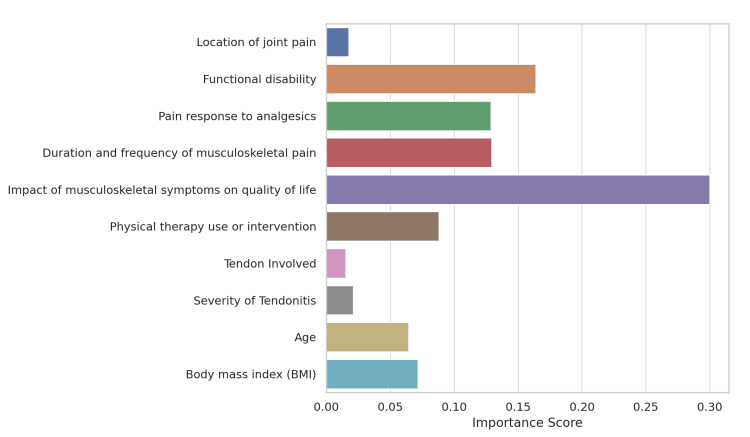
Feature importance for predicting arthralgia severity

The plot illustrates the feature importance for predicting arthralgia severity, with the impact of musculoskeletal symptoms on quality of life having the highest importance score of 0.30, followed by functional disability at 0.17 and pain response to analgesics at 0.13. Other significant predictors include the duration and frequency of musculoskeletal pain, with importance scores of 0.13 and 0.08, respectively, followed by physical therapy at 0.08, age at 0.06, and BMI at 0.07. Tendon involvement and severity of tendinitis have the lowest importance scores of 0.02 and 0.03, respectively, indicating that they contribute the least to predicting arthralgia severity compared to other clinical factors (Figure 8).

**Figure 9:**
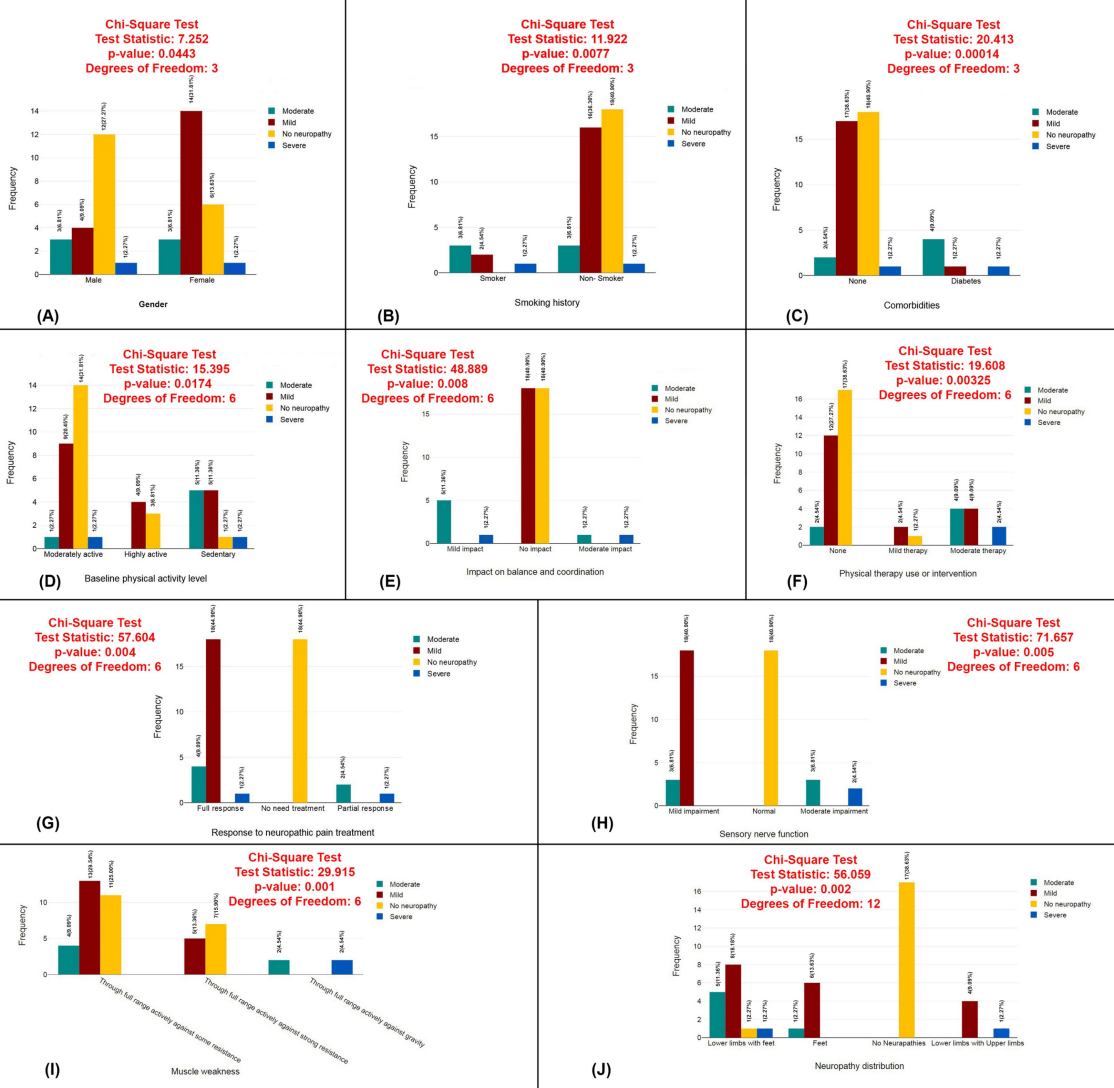
Chi-square analysis of factors associated with peripheral neuropathy severity (A) Gender distribution by peripheral neuropathy severity. (B) Smoking history by peripheral neuropathy severity. (C) Comorbidities by peripheral neuropathy severity. (D) Baseline physical activity level by peripheral neuropathy severity. (E) Impact on balance and coordination by peripheral neuropathy severity. (F) Physical therapy use or intervention by peripheral neuropathy severity. (G) Response to neuropathic pain treatment by peripheral neuropathy severity. (H) Sensory nerve function by peripheral neuropathy severity. (I) Muscle weakness by peripheral neuropathy severity. (J) Neuropathy distribution by peripheral neuropathy severity.

The plots present the results of chi-square tests assessing various factors associated with the severity of peripheral neuropathy (moderate, no neuropathy, severe). Significant relationships were found between neuropathy severity and multiple factors, including gender (χ²=7.52, p=0.0443), smoking history (χ²=11.92, p=0.0077), comorbidities (χ²=20.41, p=0.00014), baseline physical activity level (χ²=15.395, p=0.0174), balance and coordination impact (χ²=48.889, p=0.008), physical therapy use or intervention (χ²=19.608, p=0.00325), response to neuropathic pain treatment (χ²=57.604, p=0.004), sensory nerve function (χ²=71.657, p=0.005), muscle weakness (χ²=25.915, p=0.001), and neuropathy distribution (χ²=56.059, p=0.002) (Figure 9).

**Figure 10:**
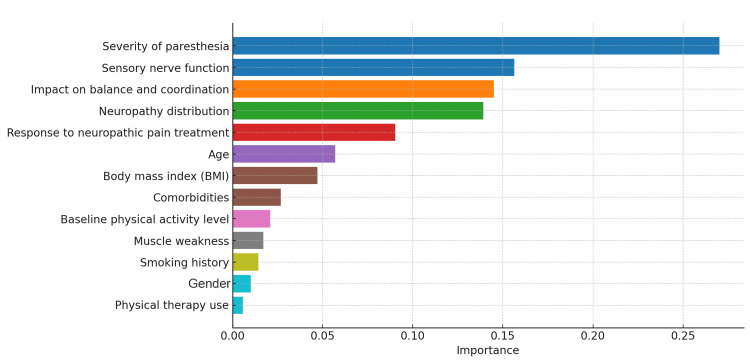
Feature importance for peripheral neuropathy severity

The plot illustrates the feature importance for predicting peripheral neuropathy severity using a random forest model, with each feature's contribution to the prediction represented along the x-axis. The most important predictor is severity of paresthesia, with an importance score of 0.27, followed by sensory nerve function at 0.16 and impact on balance and coordination at 0.14. Neuropathy distribution (0.13) and response to neuropathic pain treatment (0.08) also significantly contribute to the model. Factors such as age (0.07) and BMI (0.05) have smaller contributions. Other variables, including comorbidities, baseline physical activity level, muscle weakness, smoking history, gender, and physical therapy use, show relatively low importance scores, ranging from 0.03 to 0.01 (Figure 10).

**Figure 11:**
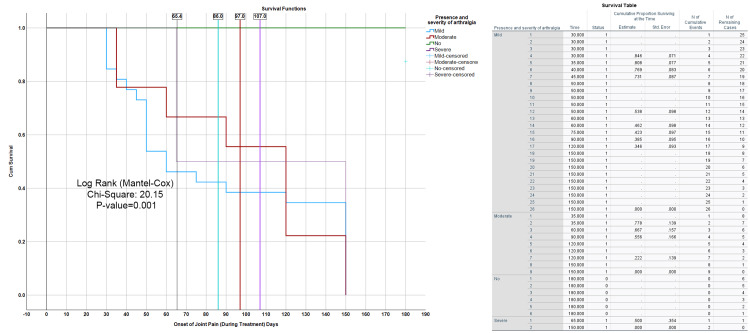
Kaplan-Meier survival curve for arthralgia severity during treatment

The Kaplan-Meier survival curve displayed in the plot illustrates the cumulative survival proportions for patients categorized by the severity of their arthralgia (joint pain), which includes mild, moderate, severe, and no pain, throughout the treatment period. Survival was measured in days, with the onset of joint pain serving as a key reference point. The log-rank (Mantel-Cox) test provides a chi-square value of 20.15 and a p-value of 0.001, suggesting a statistically significant difference in survival times among the different severity groups. The overall cumulative survival rate for all patients was 65.4 days. In contrast, patients in the mild severity group had a higher survival time, with an average of 86.0 days. The moderate severity group, however, exhibited a sharper decline in survival, beginning around day 97. Patients with severe arthralgia showed the lowest survival time, with a cumulative survival of 107 days. The accompanying survival table further elaborates on these findings, detailing the survival times and the number of remaining patients within each severity group. This supports the observed trends, highlighting how the severity of joint pain influences both survival duration and the onset of symptoms during treatment (Figure 11).

**Figure 12:**
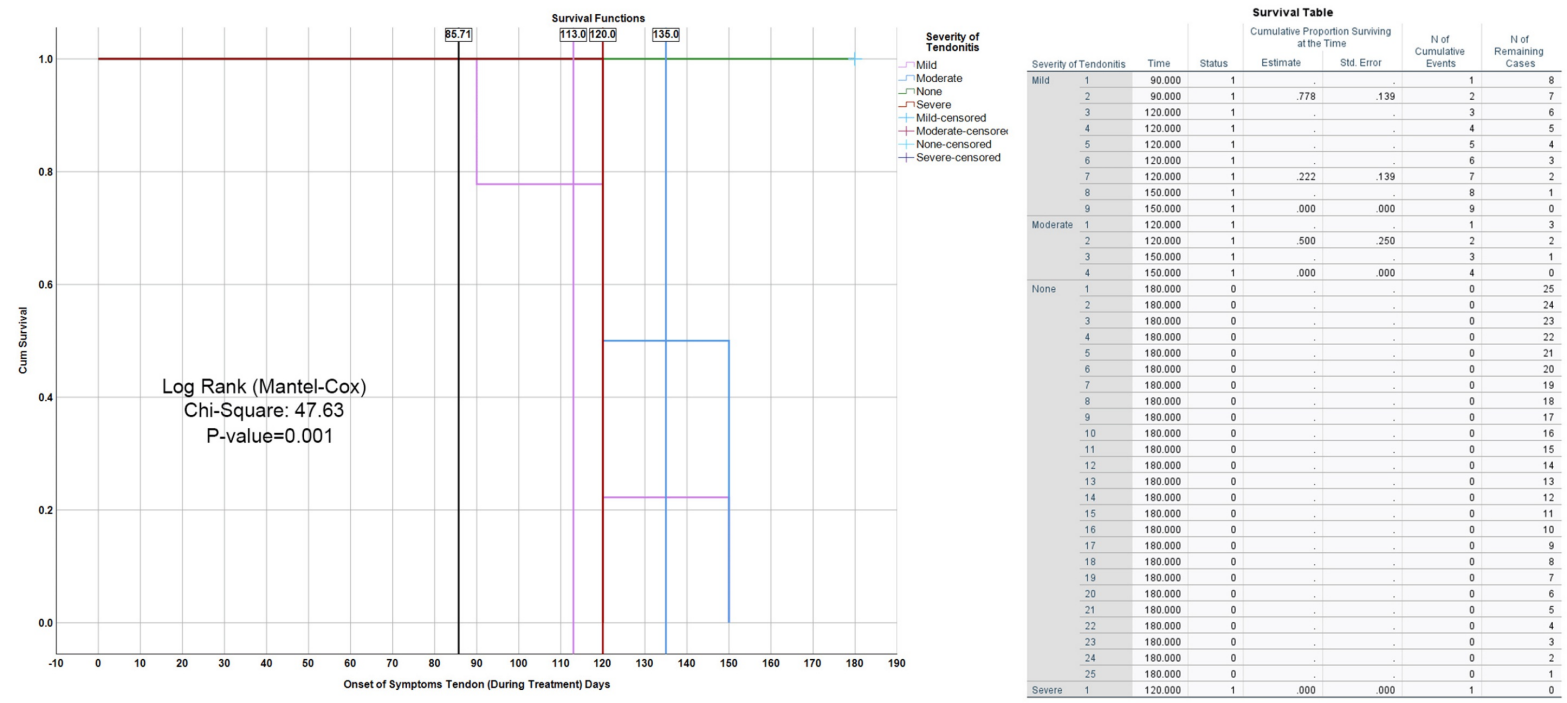
Kaplan-Meier Survival Curve for Tendonitis Severity During Treatment

The plot depicts a Kaplan-Meier survival curve for patients with varying degrees of tendonitis severity (mild, moderate, severe, and none) throughout treatment, with the onset of tendon symptoms measured in days. The survival data is analyzed using the log-rank (Mantel-Cox) test, which yields a chi-square value of 47.63 and a p-value of 0.001, suggesting a statistically significant difference in survival times among the different tendonitis severity groups. The overall cumulative survival time for all patients is recorded at 85.71 days. Patients in the mild severity group exhibit the longest survival time, with a cumulative survival of 113 days. In contrast, the moderate severity group experiences a notable decline, starting around day 135, while the severe group shows the steepest drop in survival, with most patients experiencing the onset of tendon symptoms by day 120. The None severity group, which had no tendon symptoms, maintains the highest cumulative survival rate throughout the treatment period, demonstrating no symptom onset. The survival table, located on the right, provides additional details, including the number of patients remaining at each time point within each severity group. This table reinforces the observed survival patterns and highlights the significant differences in tendonitis severity and its impact on patient outcomes during treatment (Figure 12).

**Figure 13:**
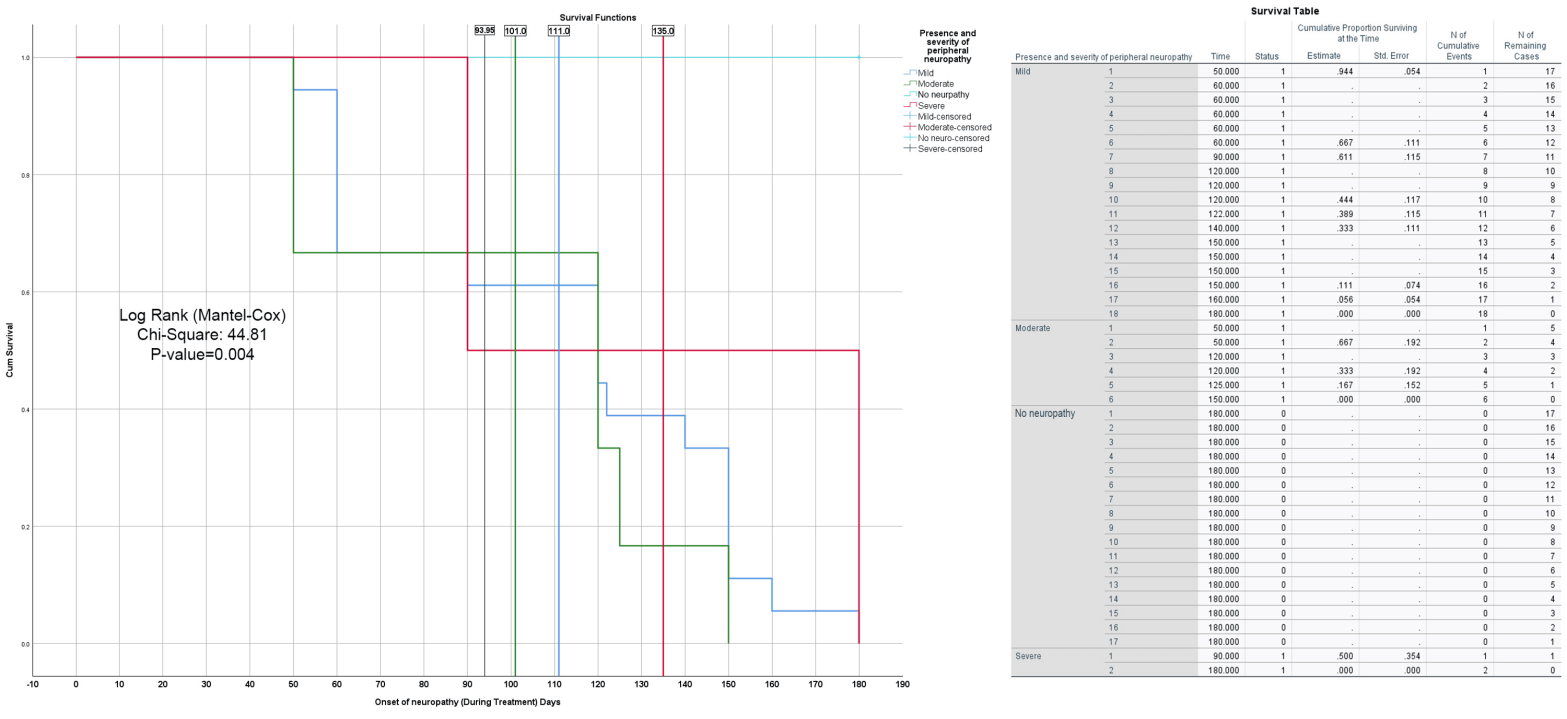
Kaplan-Meier survival curve for peripheral neuropathy severity during treatment

The plot illustrates a Kaplan-Meier survival curve for patients with varying levels of peripheral neuropathy severity (mild, moderate, severe, and no neuropathy) during treatment, with the onset of neuropathy symptoms measured in days. The data was analyzed using the log-rank (Mantel-Cox) test, which produced a chi-square value of 44.81 and a p-value of 0.004, indicating a statistically significant difference in survival times across the severity groups. The overall cumulative survival time for all patients is 93.95 days. The mild severity group shows a survival time of 101 days, while the moderate severity group demonstrates a survival time of 111 days. The severe group exhibits the sharpest decline in survival, with symptom onset occurring around day 135, leading to a significant drop in survival. In contrast, the no neuropathy group maintains a stable survival rate, showing only minimal decline throughout the treatment period. The table on the right side presents the survival data, detailing the number of patients remaining at each time point for each severity group. This table emphasizes the significant differences in survival outcomes based on the severity of peripheral neuropathy, further supporting the trends observed in the Kaplan-Meier survival curve (Figure 13).

## Discussion

This study is the first to comprehensively evaluate the clinical impact of musculoskeletal and neurological toxicities associated with the BPaLM regimen in MDR-TB patients. Despite the growing use of this regimen, limited data exist in the literature regarding its specific side effects, particularly related to musculoskeletal and neurological health. Our findings provide crucial insights into the onset, progression, and severity of arthralgia and peripheral neuropathy in a cohort of 44 MDR-TB patients, presenting a deeper understanding of these adverse effects compared to previous studies.

The demographic characteristics of our patient cohort reveal some important differences. The average age of the participants was 32.49 years, with a BMI of 17.9. This demographic profile stands in contrast to other studies involving MDR-TB populations, which tend to include older individuals or those with a higher BMI [25,26]. Additionally, our cohort had a higher proportion of females compared to males, which is a notable deviation from typical MDR-TB studies. These differences in demographics suggest that the BPaLM regimen may affect a distinct patient population, which is essential for understanding the broader impact of treatment on diverse groups.

Regarding musculoskeletal toxicity, our study found that joint pain, specifically arthralgia, typically began around day 65 of treatment. A striking 84.09% of participants experienced some degree of joint pain, with the majority reporting mild to moderate discomfort. This finding is consistent with earlier research, which noted similar musculoskeletal symptoms in MDR-TB patients receiving second-line therapy [27]. The high prevalence of knee joint pain among our participants further emphasizes the systemic nature of the side effects associated with the BPaLM regimen. These findings align with previous studies that have linked TB treatment regimens to joint and muscle discomfort, suggesting that these side effects may be an inherent part of MDR-TB treatment [28,29].

In terms of neurological toxicity, our results showed that peripheral neuropathy typically developed at an average of 93.95 days into treatment, with 59.09% of participants affected to varying degrees. This aligns with other studies highlighting linezolid, a key drug in the BPaLM regimen, as a common cause of peripheral neuropathy and other neurological side effects in MDR-TB therapy [30,31]. Additionally, our study revealed a significant relationship between prolonged treatment duration and the exacerbation of neurological symptoms, consistent with findings from other research that longer treatment periods are associated with a greater risk of neurological complications [32].

Our statistical analysis, including chi-square tests, further explored the relationship between patient demographics, physical activity levels, comorbidities, and all other variables. These analyses revealed significant correlations between higher physical activity levels and reduced severity of both arthralgia and neuropathy. This finding is particularly noteworthy, as it suggests that higher levels of physical activity may serve as a protective factor against certain treatment-related toxicities. This is consistent with literature indicating that physically active individuals may experience a buffering effect that mitigates the severity of musculoskeletal and neurological side effects associated with drug regimens [33,34]. Additionally, we observed that diabetic patients were more likely to experience severe adverse effects, which aligns with existing research indicating that diabetes can exacerbate the risks of drug-induced complications [35,36].

In terms of managing treatment-related pain, our study found that a significant proportion of patients (75%) experienced complete relief from pain through the use of analgesics and neuropathic pain treatments. This aligns with previous studies that have reported mixed results regarding the efficacy of pain management strategies in TB patients, with some showing promising outcomes and others reporting suboptimal relief [37,38]. However, it is important to note that failure to properly report and manage adverse events can lead to noncompliance with treatment and hinder patient recovery, as highlighted by several studies on the challenges of managing TB treatment toxicities [39].

The impact of musculoskeletal and neurological symptoms on patients' quality of life is another critical aspect of our study. We found that 72.73% of patients did not experience functional disability related to joint pain, which suggests that alternative pain management strategies or timely interventions may be effective in mitigating the impact of these symptoms. However, the psychological toll of chronic pain and its potential influence on treatment adherence should not be overlooked. Other studies have demonstrated a significant link between chronic pain and psychological distress in TB patients, emphasizing the need for integrated mental health support alongside physical symptom management [40,29].

Our decision tree model analysis further identified key predictive factors for the severity of arthralgia and peripheral neuropathy, underscoring the importance of early demographic assessments in personalizing treatment regimens. This finding supports the growing body of literature advocating for personalized medicine in the management of MDR-TB, where tailored treatment approaches could help reduce both the physical and psychological burdens of treatment [41-43].

Overall, our study provides valuable insights into the clinical toxicities of the BPaLM regimen in MDR-TB patients, contributing to a limited but critical body of knowledge. These findings underscore the importance of early detection, monitoring, and management of musculoskeletal and neurological side effects to improve patient outcomes and quality of life during MDR-TB treatment.

While this study provides valuable insights into the prevalence and severity of musculoskeletal and neurological toxicities associated with the BPaLM regimen, several limitations should be acknowledged. First, the study was conducted with a relatively small sample size of 44 patients, which may limit the generalizability of the findings. Future studies with larger sample sizes and multi-center involvement are needed to confirm these results and provide a more comprehensive understanding of the BPaLM regimen's impact on diverse populations. Additionally, this study primarily focused on the short-term effects of the BPaLM regimen, and a longer-term follow-up period is required to fully assess the durability of musculoskeletal and neurological toxicities over time. As the long-term impact of treatment-related side effects remains unclear, future research should aim to investigate how these toxicities evolve over extended treatment durations and after treatment completion.

Furthermore, this study did not explore the molecular mechanisms underlying the development of arthralgia and peripheral neuropathy, which could offer deeper insights into the pathophysiology of these adverse effects. Future research should investigate these mechanisms at the biochemical and genetic levels to enhance our understanding of why certain patients develop more severe symptoms than others. Moreover, convenience sampling was employed to select participants, which may introduce selection bias. This approach could limit the external validity of the study's findings, as the sample may not fully represent the broader MDR-TB population. A more randomized sampling method would strengthen the reliability of the results and enhance their generalizability to diverse patient groups. Lastly, the study relied on self-reported pain and symptom severity, which could introduce subjectivity and bias into the results. While these measures are commonly used in clinical settings, future studies should consider incorporating objective assessments, such as biochemical markers or imaging techniques, to validate the severity of arthralgia and neuropathy.

## Conclusions

This study contributes significantly to the understanding of musculoskeletal and neurological toxicities associated with the BPaLM regimen in MDR-TB patients. The findings indicate that both arthralgia and peripheral neuropathy are common and can vary in severity, impacting patients' quality of life and treatment adherence. Key clinical factors, including age, BMI, and physical activity level, were identified as important predictors of toxicity severity. The study highlights the importance of early monitoring, symptom management, and intervention strategies, including the use of analgesics and physical therapy, to mitigate the adverse effects. Further research is needed to investigate the long-term effects of these toxicities and the molecular mechanisms underlying their development, as well as the efficacy of potential interventions.
